# Results of using robotic-assisted navigational system in pedicle screw placement

**DOI:** 10.1371/journal.pone.0220851

**Published:** 2019-08-19

**Authors:** Hsuan-Yu Chen, Xiu-Yun Xiao, Chih-Wei Chen, Hao-Kai Chou, Chen-Yu Sung, Feng-Huei Lin, Po-Quang Chen, Tze-Hong Wong

**Affiliations:** 1 Department of Orthopedic Surgery, National Taiwan University Hospital, Hsin-Chu branch, Hsinchu City, Taiwan; 2 Department of Biomedical Engineering, National Taiwan University, Taipei City, Taiwan; 3 Point Robotics MedTech Inc., Hsinchu City, Taiwan; University Magna Graecia of Catanzaro, ITALY

## Abstract

Recent technical developments have resulted in robotic-assisted pedicle screw placement techniques. However, the use of robotic-assisted navigational techniques is still subject to controversy. This study aims to assess the accuracy and safety of a self-developed navigation system, the point spine navigation system (PSNS), for robotic-assisted pedicle screw placement surgery. Fifty-nine pedicle screws were implanted in three porcine vertebrae at the T6–T10 and L1–L5 levels, with the assistance of the PSNS. The navigation and planning system provides virtual surgical guide images, including sagittal, coronal, axial, oblique planes, and customized three-dimensional reconstructions for each vertebra to establish accurate pedicle screw trajectories and placement tracts. After pedicle screw placement, post-operative spiral computer tomographic scans were performed and screws were evaluated using the Gertzbein–Robbins classification. Differences between the actual pedicle screw position and pre-operative planning paths, including the angle, shortest distance, and entry trajectory were recorded. The 59 pedicle screw placements were all within a safe zone, and there was no spinal canal perforation or any other damage under postoperative computed tomography image data. Fifty-one screws were categorized as group A, seven screws were noted as group B, and one screw was identified as group E under the Gertzbein–Robbins classification. The mean entry point deviation was 2.71 ± 1.72°, mean trajectory distance was 1.56 ± 0.66 mm, and average shortest distance between two paths was 0.96 ± 0.73 mm. Pedicle placement remains a challenging procedure with high reported incidences of nerve and vascular injuries. The implementation of a robotic-assisted navigational system yields an acceptable level of accuracy and safety for the pedicle screw placement surgery.

## Introduction

Free-handed pedicle screw placement remains a high-risk procedure owing to the many important structures near the pedicle, such as the spinal cord, nerve root, and associated vessels, despite the advancements in fluoroscopy [1]. Complications from such procedures include neurological deficits, vascular injuries associated with incorrect positioning of pedicle screw, or screw loosening after screw repositioning [1, 2]. Moreover, radiation exposures for the surgeon and staff increase, particularly in minimally invasive spinal procedures [3]. Recent technical developments have resulted in robotic-assisted pedicle screw placement techniques [4]. However, the use of a robotic-assisted navigational system is still subject to controversy [5]. The aim of this study is to assess the accuracy and safety of a self-developed navigation system, the point spine navigation system (PSNS), for robotic-assisted pedicle screw placement surgery.

## Materials and methods

### Point spine navigation system (PSNS)

**Fig 1:**
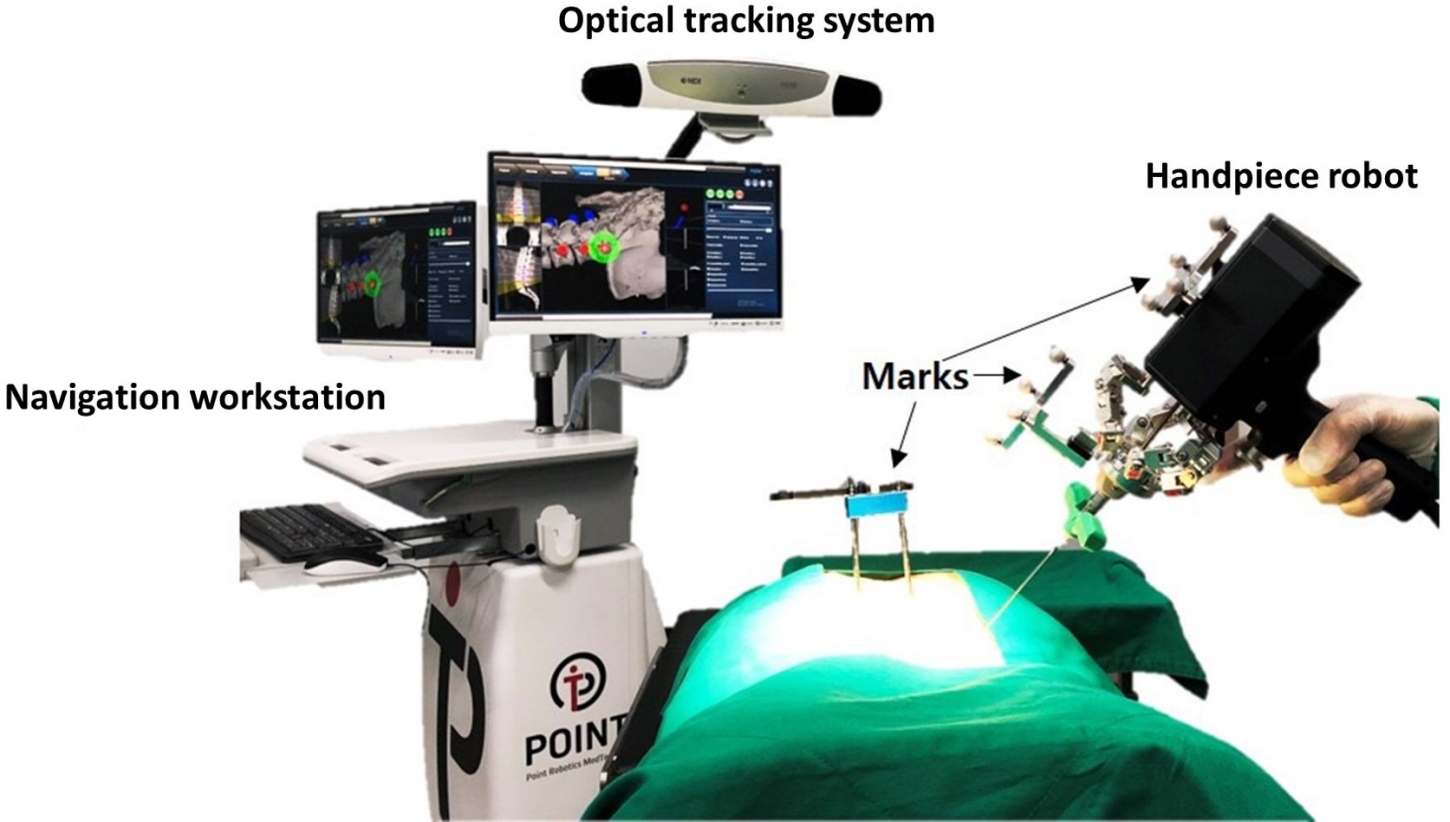
Point spine navigation system (PSNS).

The PSNS consists of a navigation workstation (optical tracking system, computer, monitor, cart, navigation and planning systems, and handpiece robot control unit); handpiece robot (a surgical robot equipped with six degrees of freedom, DOF), and navigational instruments (probe, PSNS fiducial frame—FF). Each part of the system was designed with the workspace availability and necessity for operation in mind (Fig 1).

### Navigation workstation

The navigation and planning system provides virtual surgical guide images, including sagittal, coronal, axial, oblique planes, and customized three-dimensional reconstructions for each vertebra to establish and transfer the pedicle screw trajectories and directions to the computer monitor. Using an optical tracking system, we can match the porcine anatomy with pre-operative planning images. After completing the planned surgical path, this information was transferred to the handpiece robot by the handpiece robot control unit. Intraoperatively, the navigation system was also able to integrate the tracking information and display it on a monitor in real time. Further, the admission path on the spine and the real-time positions of the instruments were also displayed on the screen.

### Tracking system

The infrared optical tracking system comprised a camera (NDI, Vega, USA), passive markers, and probe. The markers were attached to the robot and vertebra to be navigated, and the probe was used to define the point coordinates in space. With these markers, the tracker can monitor the positions and movements of the robot end-effector in real-time. Calibrations must be performed before commencing operation. We used the handpiece robot to create a variety of gestures and to record the kinematics of the robot through the optical tracking system to create a precise set of kinematic parameters. For the registration in the PSNS system, we selected fiducial points (reference pins) on the image displayed and selected the corresponding positions of the points using the probe in real-world coordinates. Through repeated selections and measurements, the coordinate frame of the image and that of the real porcine body were synchronized. We fixed eight markers on the PSNS handpiece robot, which were tracked by the tracking device. After registration, the positions and orientations of the markers were converted to the positions and orientations of the handpiece robot.

### Handpiece robot

The handpiece robot was composed of a 6-DOF Stewart platform, and the end of the robot was equipped with an operation tool used for drilling the screw path. Intraoperatively, the movement of the surgical target is tracked in real-time, and the robot automatically compensates for the correct target.

### Inclusion and exclusion criteria

The pedicles of the experimental porcine model were evaluated to ensure that they were intact through preoperative computed tomography (CT) scans. The pedicle screws selected were 4.5 mm in diameter and 30 mm in length, which is a feasible size. Incomplete and narrow pedicles were excluded.

### Surgical outflow

**Fig 2:**
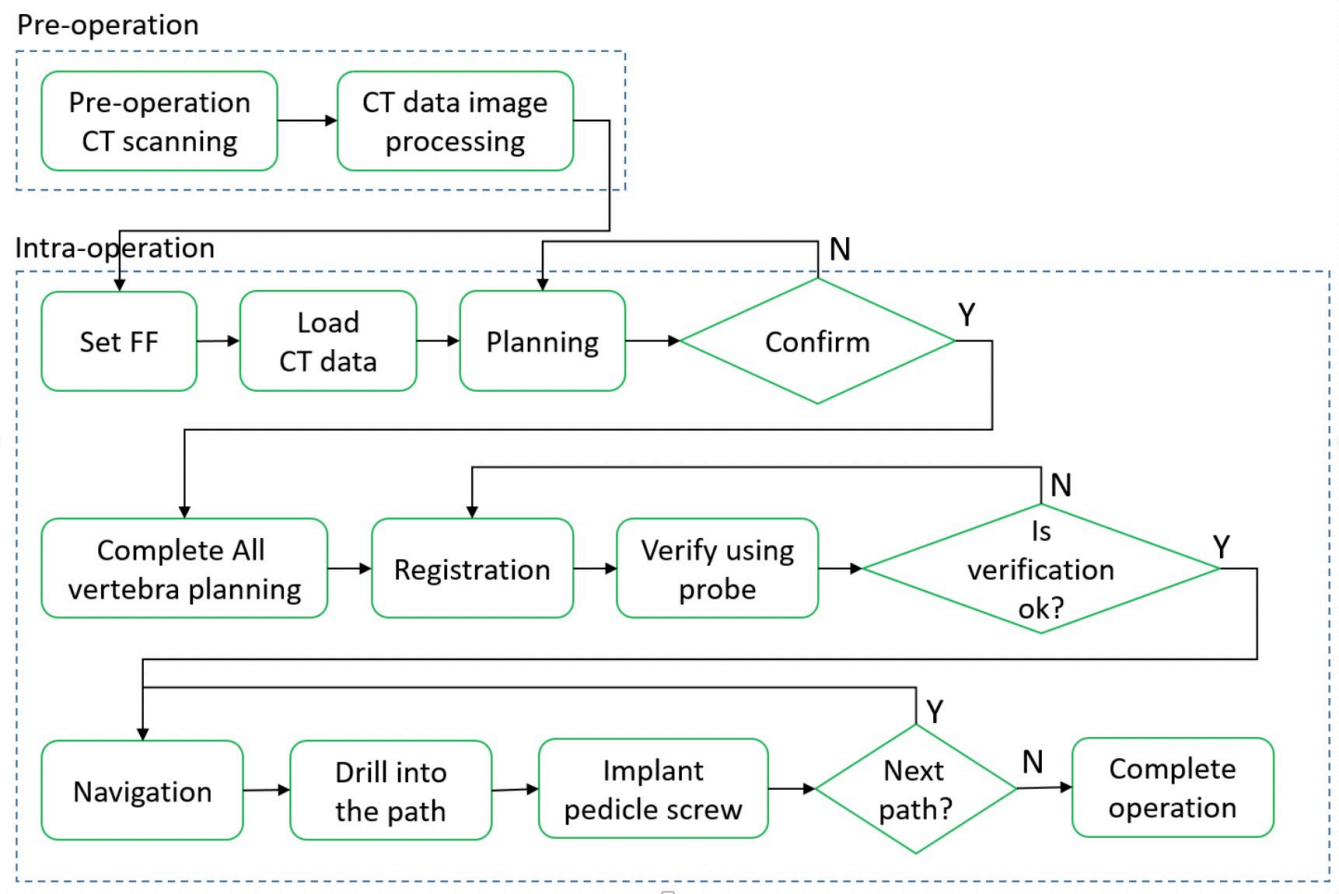
Procedure of screw placement operation using the PSNS.

As shown in Fig 2, the surgical workflow was mainly divided into two stages. In the pre-operation stage, the reference pins were fixed onto the porcine vertebrae and were scanned by CT. The CT data can be loaded into the PSNS system after image processing. In the intraoperation stage, the surgeon attaches the markers on the porcine vertebrae. After loading the CT images into the PSNS system, the surgeon can perform surgical planning and confirmation through the system under three-dimensional bone model reconstruction. After using the probe to select the reference pin to confirm registration, the surgeon can move the handpiece robot close to the entry point. The robot accurately adjusts to the planned trajectory and has a compensation function. After being checked by the surgeon, the robot begins to drill into the vertebra. The navigation information will be displayed on the monitor in real time. The robot stops drilling at a set end point, and the robot motion is similarly repeated for all the required implants. The surgeon then inserts the screws to complete the operation.

### Porcine spine experiment

**Fig 3:**
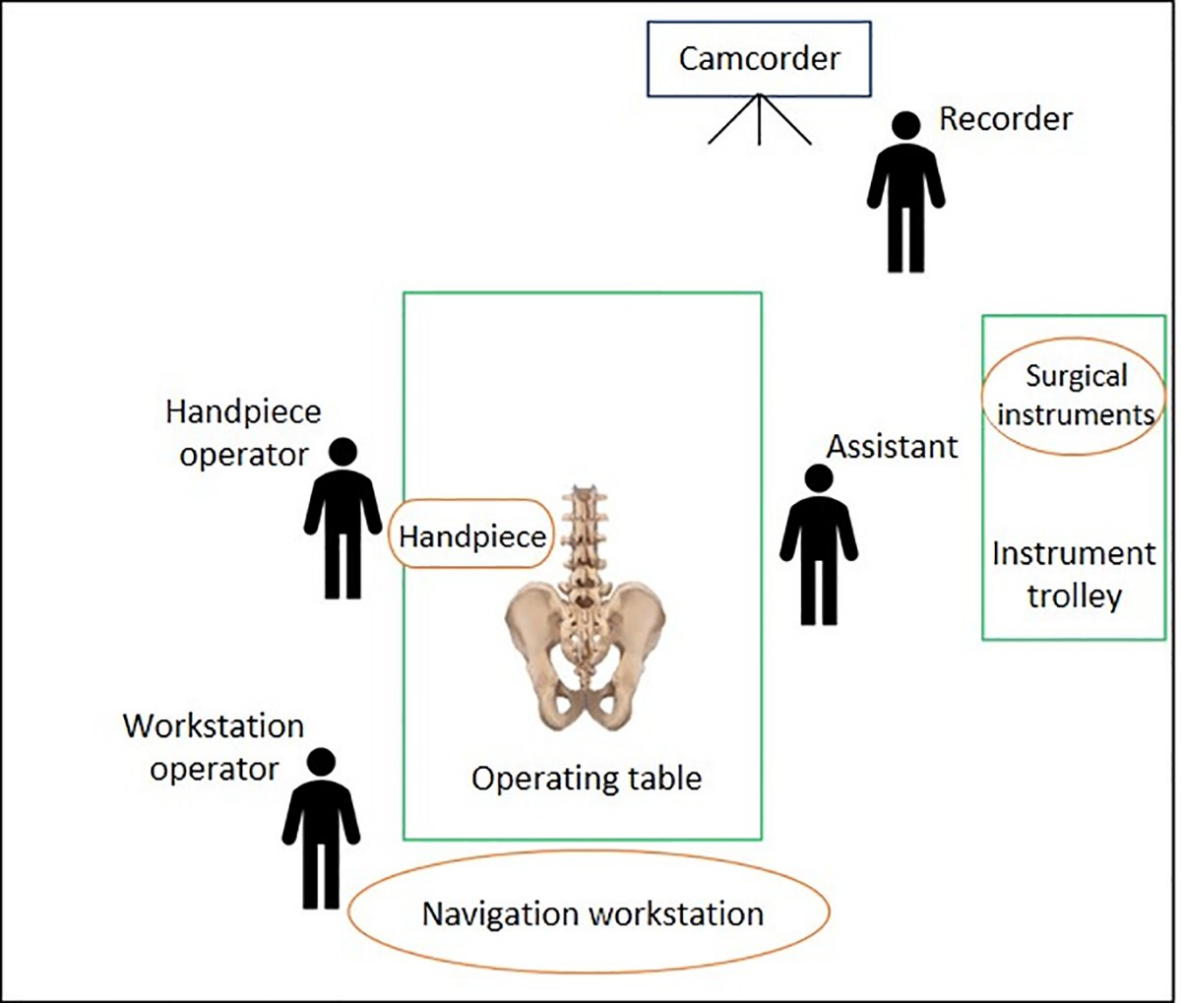
Experimental setup with a porcine spine.

A total of three porcine vertebrae at the T6–T10 and L1–L5 levels were used, and the porcine spines used in our study was procured from National Taiwan University and approved by the Certified Agricultural Standards (CAS) of Taiwan. The porcine spines were immobilized on the operating table in the prone position, and the procedure was performed as described above (Fig 3).

### Evaluation

After the operation, postoperative CT scans weres performed. Standard measurements based on postoperative CT scans and acknowledged criteria, the Gertzbein–Robbins classification [6], were adopted to evaluate the inserted pedicle screws by a unified accuracy measure. The position of the screw on the pedicle was measured in millimeters and did not cause any damage so long as it was within the safe range in this study.

## Results

**Fig 4:**
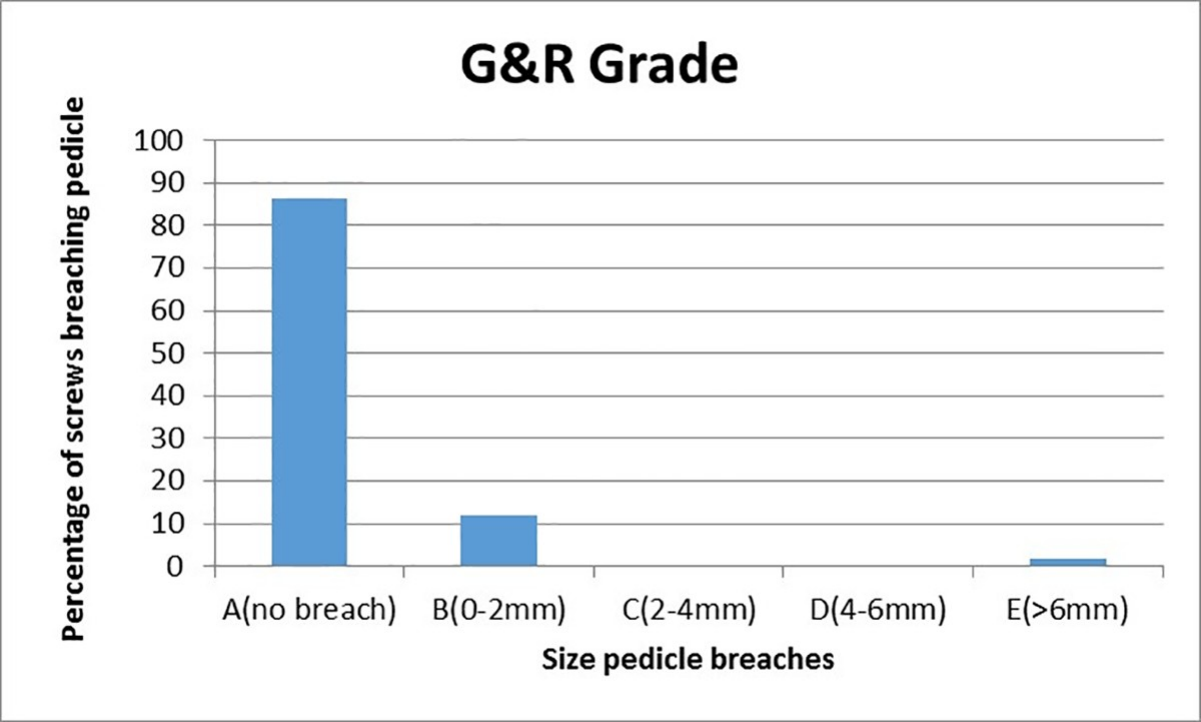
Column chart showing the percentage of screws breaching the pedicle wall according to the Gertzbein–Robbins criterion. Of all the 59 robotic-guided screws, 98.3% (58) were safely placed (Groups A or B), whereas 1.7% (1) breached the intrapedicular trajectory (Group E).

**Fig 5:**
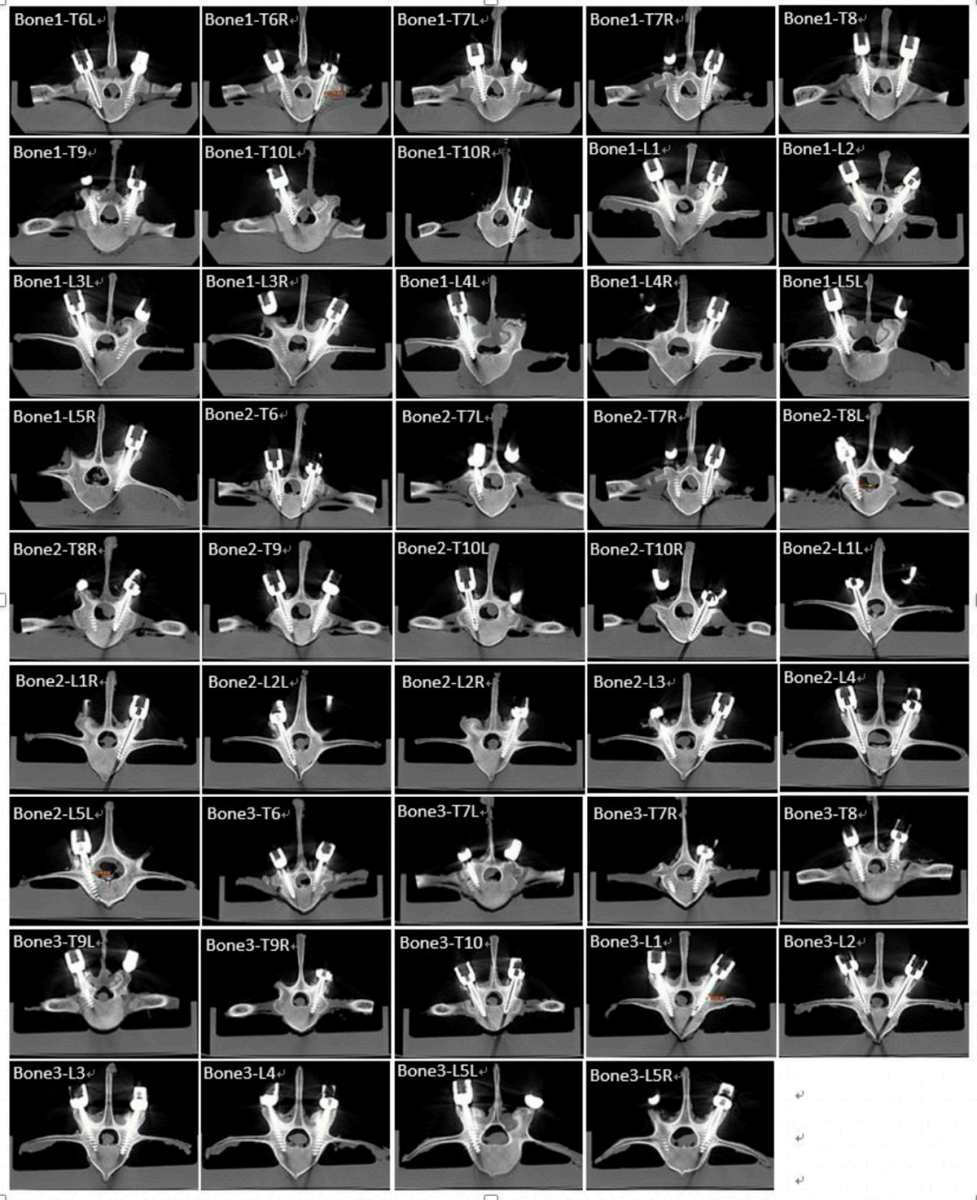
Screw position accuracy measurements determined from postoperative CT scans.

A total of 60 screws were placed in 30 vertebrae, and one of the screw placements was excluded from analysis owing to robotic guidance abortion for unknown reason. This study demonstrated a low overall screw malposition rate of 1.7% for robotic-assisted screw placements, and the deviations were assessed by postoperative CT scans. For the remaining 59 pedicle screws, surgical procedures were smoothly performed using the PSNS. According to the Gertzbein–Robbins classification, 51 screws (86.4%) fell into group A, 7 screws (11.9%) fell into group B, and 1 screw (1.7%) fell into group E (Fig 4). All pedicle screws were inserted within the safe zone, and there were no spinal canal perforations or injuries to any other major vessels (Fig 5) [7].

**Fig 6:**
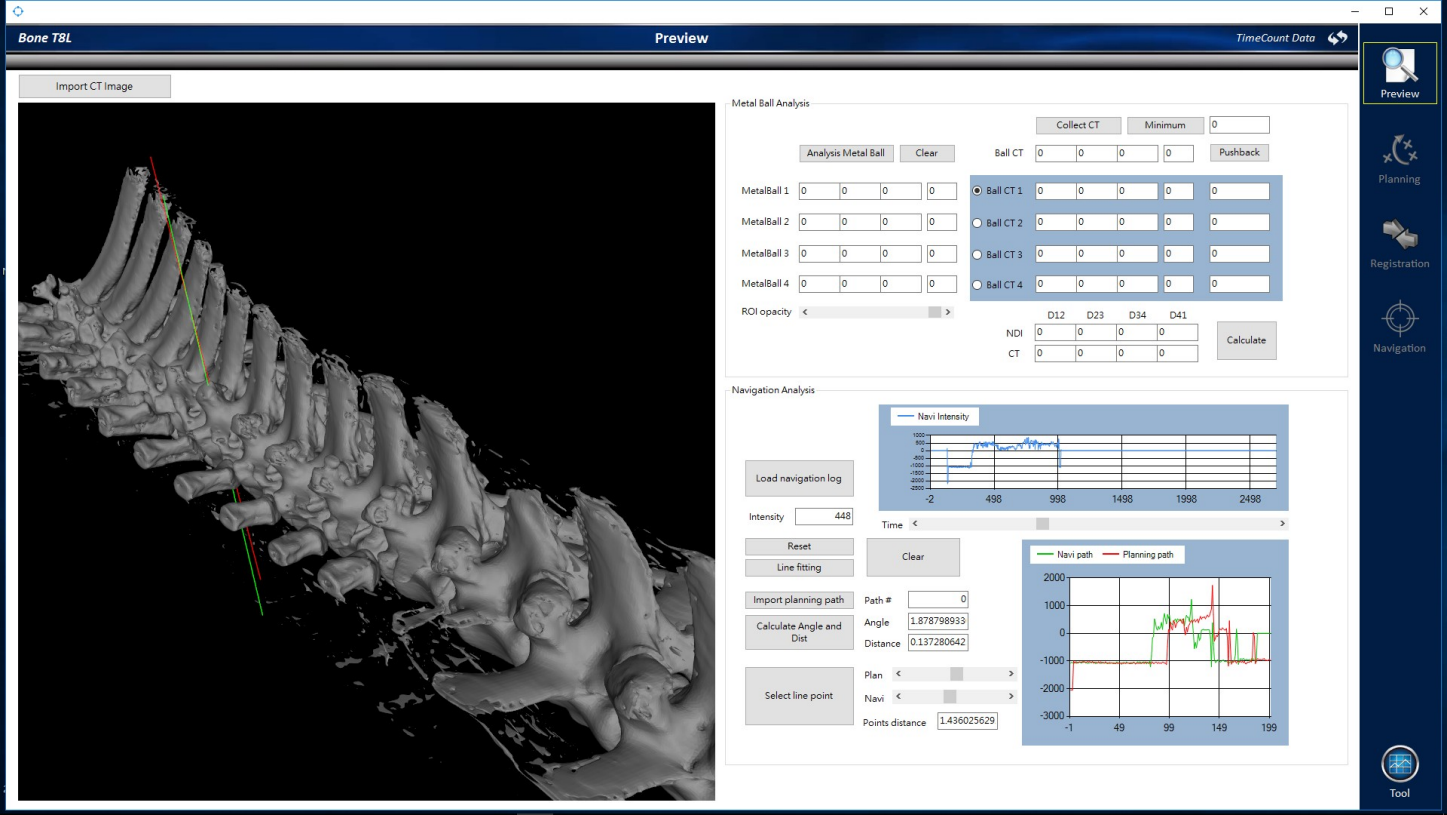
Difference between the actual pedicle screw movement position and preoperative planning path.

**Fig 7:**
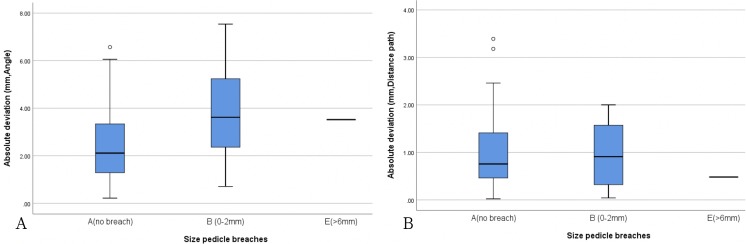
Boxplot showing correlations between actual pedicle screw positions and preoperative planning paths for different groups.

During the surgery, the optical tracking system was used to record the tip position data at a frequency of 60 Hz, and a linear regression curve was calculated. Differences between the actual pedicle screw position (green line) and preoperative planning path (red line), including the angle, shortest distance, and entry point, were also recorded (Fig 6). The mean entry-point deviation was 2.71 ± 1.72degree, mean trajectory distance was 1.56 ± 0.66 mm, and average shortest distance between the two paths was 0.96 ± 0.73 mm (Table 1). Further analyses were performed according to the Group A, B, and E classifications. The boxplots in Fig 7 show the correlations between the actual pedicle screw positions and preoperative planning paths for the different groups. The mean entry point deviations of these three groups (in degree) are as follows: Group A, mean = 2.536, medium = 2.1150, max = 6.57, min = 0.22; Group B, mean = 3.8686, medium = 3.62, max = 7.54, min = 0.71; Group E—3.52 (Fig 7A). The mean shortest distance paths (in mm) are as follows: Group A, mean = 0.9706, medium = 0.7550, max = 3.39, min = 0.02; Group B, mean = 0.9614, medium = 0.9100, max = 2.00, min = 0.04, Group E—0.48 (Fig 7B).

**Table 1:** Comparison between actual pedicle screw position and preoperative planning path.

Porcine Spine	Angle(degree) Mean ± SD	Distance of entry point (mm) Mean ± SD	Distance of path (mm) Mean ± SD
1 (screws placed = 20)	2.57±1.51	1.41±0.84	0.76±0.70
2 (screws placed = 19)	2.92±1.96	1.61±0.55	1.04±0.81
3 (screws placed = 20)	2.65±1.71	1.66±0.57	1.07±0.68
Total (screws placed = 59)	2.71±1.72	1.56±0.66	0.96±0.73

## Discussion

Surgical robotics applications have developed rapidly since the 1990s to optimize the available robotic technologies, and they are designed to enhance and complement the surgeon’s abilities [8]. Surgeons may experience fatigue and hand tremors after lengthy and tedious spinal surgery procedures, including screw placements, bone osteotomy, and nerve decompression [9]. Spinal fusion is the foundation of spinal surgeries, and pedicle screws provide substantial rigidity to facilitate fusion. Pedicle screw placements have achieved widespread use in the surgical treatment of different spinal diseases and conditions, such as degenerative, traumatic, and developmental spinal conditions [1]. Free-hand pedicle placement techniques depend largely on the anatomic landmarks, image guides, and the surgeon’s experience. Even with experienced surgeons, the implant malposition rates range from 5.1% to 31%, as described in multiple review studies [2, 10].

There have been several debates regarding the safety and accuracy of robotic-assisted pedicle screw placements [11, 12]. In our study, the ideal screw trajectory was planned preoperatively with a CT-based navigation system; these images could then be accurately matched to porcine anatomy during surgery using optical tracking. Intraoperatively, the navigation system was also able to integrate tracking information for real-time display on a monitor. The handpiece robot control unit was able to completely avoid the facets of the vertebra without compromising screw purchase within the pedicle, and the robotic guidance system was able to reproduce the planned path almost accurately regardless of the surgeon’s proficiency during operation. However, we found that there was a higher rate of laterally misplaced screws during such procedures. One explanation for this may be an unconscious tendency to plan trajectories more laterally to avoid contact with the spinal canal. One of the other possible sources of inaccuracy is the phenomenon of a cannula sliding off the facet joint. We use three measures to improve accuracy in the study. First, the trajectory of the pedicle screw was prepared by high-speed drilling instead of a hammer to prevent lateral skidding. Second, we chose a lateral-to-medial trajectory to minimize lateral skidding. Finally, the upgraded system allows better visualization, which may have contributed to the improved accuracy. In one case, there was lateral wall breaching with a screw, and no neurologic deficit, weak maximal screw insertional torque, compromised seating torque, or weak axial pullout strength were associated with this incident.

Differences between the actual pedicle screw position (green line in Fig 5) and preoperative planning path (red line in Fig 5), including the angle, shortest distance, and entry trajectory, were within normal ranges. Interestingly, we observed that two of our pedicle screws were placed in better positions than those planned. One possible reason for this could be the guide pin procedure that we used; once the guide pin was in the pedicle, after tapper and screw placement, the hard cortex part would automatically adjust the pedicle screw into a better position.

Deviation of the trajectory could be attributed to soft tissue pressures, forceful surgical application, and bony lateral skidding. Further applications to percutaneous cases and optimal point of entry at the skin level are easy to achieve with robotic platforms without muscle resistance owing to the transmuscular approach, whereas free-hand techniques work against strong tissue resistance, and robotic-guided procedures require significantly shorter imaging sessions and radiation exposure [13]. The use of fluoroscopic control throughout the learning curve was suggested to maintain control over the proposed trajectory.

### Study limitation

Our study had certain limitations for a small number of pedicle screw placements without soft tissue involvement. Traditional methods defining the entry point using both visual anatomical landmarks and navigation pointers simultaneously might strengthen the accuracy of pedicle screw placement in our work. This should be confirmed on a larger data sample; further studies on percutaneous procedures are thus planned to validate the application of this tool in spinal surgery.

## Conclusion

Robotic-assisted pedicle screw placements are encouraging for surgical procedures and have a high accuracy of up to 98.3%. We believe that this study has proven the potential for improving surgical outcomes with robotic-assisted systems despite the early stage at which robotic surgery currently stands, especially where complicated operations and minimal invasiveness are required. These findings demonstrate that the implementation of the point spine navigation system (PSNS) improves accuracy of pedicle screw placements and ensures optimal safety of pedicle screw placement surgery.

## References

[1] Gaines RW Jr. The use of pedicle-screw internal fixation for the operative treatment of spinal disorders. J Bone Joint Surg Am. 2000;82-a(10):1458–76. 10.2106/00004623-200010000-00013 11057475

[2] Dede O, Ward WT, Bosch P, Bowles AJ, Roach JW. Using the freehand pedicle screw placement technique in adolescent idiopathic scoliosis surgery: what is the incidence of neurological symptoms secondary to misplaced screws? Spine (Phila Pa 1976). 2014 2 15;39(4):286–90. 24553446 10.1097/BRS.0000000000000127

[3] Erratum: Radiation exposure in spine surgery using an image-guided system based on intraoperative cone-beam computed tomography: analysis of 107 consecutive cases. J Neurosurg Spine. 2017;26(4):542 10.3171/2016.11.SPINE151139a 28084930

[4] Kim TT, Johnson JP, Pashman R, Drazin D. Minimally Invasive Spinal Surgery with Intraoperative Image-Guided Navigation. Biomed Res Int. 2016;2016:5716235 10.1155/2016/5716235 27213152 PMC4860212

[5] Kosmopoulos V, Schizas C. Pedicle screw placement accuracy: a meta-analysis. Spine (Phila Pa 1976). 2007 2 1;32(3):E111–20. 17268254 10.1097/01.brs.0000254048.79024.8b

[6] Gertzbein SD, Robbins SE. Accuracy of pedicular screw placement in vivo. Spine (Phila Pa 1976). 1990 1;15(1):11–4. 2326693 10.1097/00007632-199001000-00004

[7] Pechlivanis I, Kiriyanthan G, Engelhardt M, Scholz M, Lücke S, Harders A, et al Percutaneous placement of pedicle screws in the lumbar spine using a bone mounted miniature robotic system: first experiences and accuracy of screw placement. Spine (Phila Pa 1976). 2009 2 15;34(4):392–8. 19214099 10.1097/BRS.0b013e318191ed32

[8] Kantelhardt SR, Martinez R, Baerwinkel S, Burger R, Giese A, Rohde V. Perioperative course and accuracy of screw positioning in conventional, open robotic-guided and percutaneous robotic-guided, pedicle screw placement. Eur Spine J. 2011 6;20(6):860–8. 10.1007/s00586-011-1729-2 21384205 PMC3099153

[9] Stüer C, Ringel F, Stoffel M, Reinke A, Behr M, Meyer B. Robotic technology in spine surgery: current applications and future developments. Acta Neurochir Suppl. 2011;109:241–5. 10.1007/978-3-211-99651-5_38 20960350

[10] Bailey SI, Bartolozzi P, Bertagnoli R, Boriani S, van Beurden AF, Cross AT, et al The BWM spinal fixator system. A preliminary report of a 2-year prospective, international multicenter study in a range of indications requiring surgical intervention for bone grafting and pedicle screw fixation. Spine (Phila Pa 1976). 1996 9 1;21(17):2006–15. 8883203 10.1097/00007632-199609010-00016

[11] Devito DP, Kaplan L, Dietl R, Pfeiffer M, Horne D, Silberstein B, et al Clinical acceptance and accuracy assessment of spinal implants guided with SpineAssist surgical robot: retrospective study. Spine (Phila Pa 1976). 2010 11 15;35(24):2109–15. 21079498 10.1097/BRS.0b013e3181d323ab

[12] Roser F, Tatagiba M, Maier G. Spinal robotics: current applications and future perspectives. Neurosurgery. 2013 1;72 Suppl 1:12–8. 23254800 10.1227/NEU.0b013e318270d02c

[13] Lieberman IH, Hardenbrook MA, Wang JC, Guyer RD. Assessment of pedicle screw placement accuracy, procedure time, and radiation exposure using a miniature robotic guidance system. J Spinal Disord Tech. 2012 7;25(5):241–8. 10.1097/BSD.0b013e318218a5ef 21602728

